# Persistence of dengue genome in a remotely infected patient

**DOI:** 10.2478/abm-2023-0072

**Published:** 2023-12-28

**Authors:** Soraya Thaivanich, Jirayu Visuthranukul, Kesinee Arunyingmongkol, Udomsak Bunworasate, Padet Siriyasatien, Wanla Kulwichit

**Affiliations:** Clinical Excellence Center for Emerging Infectious Diseases, King Chulalongkorn Memorial Hospital, Thai Red Cross Society, Bangkok 10330, Thailand; Department of Medicine, Faculty of Medicine, Chulalongkorn University, Bangkok 10330, Thailand; Department of Parasitology, Faculty of Medicine, Chulalongkorn University, Bangkok 10330, Thailand

**Keywords:** dengue, G-CSF, hematopoietic, pathogenesis, persistence, serotype, stem cell person with remote infection

## Abstract

**Background:**

Dengue virus infection is an intriguing illness. It is traditionally thought of as a self-limited and nonpersistent disease.

**Objectives:**

We report a case with persistent dengue virus genome detectable in hematopoietic cells of a person with remote infection.

**Methods:**

A patient with multiple myeloma in remission was prepared for peripheral blood stem cell (PBSC) transplantation. Plasma and G-CSF-stimulated, mobilized PBSCs were collected. Dengue-specific reverse transcription polymerase chain reaction (RT-PCR) was performed in both pre- and post-stimulated blood specimens. Anti-dengue antibodies by ELISA and by neutralization assay were measured before and after the stem cell mobilization.

**Results:**

The viral genome was detected only in the PBSC of the post-G-CSF-stimulated specimens. Anti-dengue antibodies were negative and positive, by ELISA and neutralization assays, respectively, both before and after stem cell mobilization.

**Conclusion:**

Our findings reveal a persistent infection. Whether and how this strain may interact with subsequent serotype(s) remains to be elucidated.

Dengue infection is one of the most important arthropod-borne illnesses worldwide, with clinical severity ranging from asymptomatic infection to shock and death [1]. There are 4 serotypes of the virus (DENV-1 to DENV-4), which is transmitted among humans mostly by *Aedes aegypti* mosquitoes. Primary infection by each of these serotypes is expected to provide life-long immunity for that serotype. Secondary infection occurs when one is infected for the second time by a different serotype. The degree of “immune enhancement” is widely believed to be a part contributing to severity of dengue cases. However, while almost all cases with severe serum leakage and shock are those of secondary infection, most secondary cases are ironically either asymptomatic or mild. Thus, more explanation on pathogenesis of severe dengue infection is certainly needed.

Dengue virus, perhaps similar to influenza virus and enteroviruses, is not traditionally considered to be persistent in human hosts. Rather, they are believed to cause only acute infection and are cleared by the immune system of infected individuals. Only by different serotypes could the virus reinfect the individuals for several times. Dengue virus has tropism for hematopoietic cells, and thus, if the virus can persist in human hosts, the genome may be detectable in bone marrow-derived cells. Our preliminary study, using bone marrows from hematologic malignancy patients in remission, has supported this persistence phenomenon by detecting dengue genome in 6 of 83 patients [2]. Here, we report a case of myeloma patient in remission who has evidence of persistent dengue genome from a remote infection. The patient has given written informed consent to publish this report.

## Case report

A 54-year-old woman with a prior history of treated multiple myeloma had been in disease remission for 3 years. She became a candidate for autologous peripheral blood stem cell (PBSC) transplantation. She had no clinical history of dengue diseases. Intravenous granulocyte colony-stimulating factor (G-CSF) was given to stimulate hematopoietic stem cells to enter peripheral blood for collection, per the protocol of the National Stem Cell Donor Registry Program, Thai Red Cross Society. We collected blood samples both before and 2 weeks after G-CSF stimulation, for paired anti-dengue antibodies and for detection of dengue genome before and after PBSC stimulation. This was performed after obtaining approval from the Institutional Review Board of the Faculty of Medicine (certificate of approval No. 551/2009) and after obtaining written informed consent from the volunteer. Paired anti-dengue antibody titers were performed by ELISA [3] with a minor modification and by a focus reduction neutralization test (FRNT), as previously described [4]. The stem cell collection process was carried out at the National Stem Cell Registry Program, Thai Red Cross Society, per their standard protocols.

Anti-dengue antibodies by ELISA in this case were below cut-off levels for both samples, indicating no evidence of recent dengue infection. However, as expected, the FRNT in both samples demonstrated significantly detectable immunity to dengue virus, as seen in almost all Thai adults.

**Figure 1.**
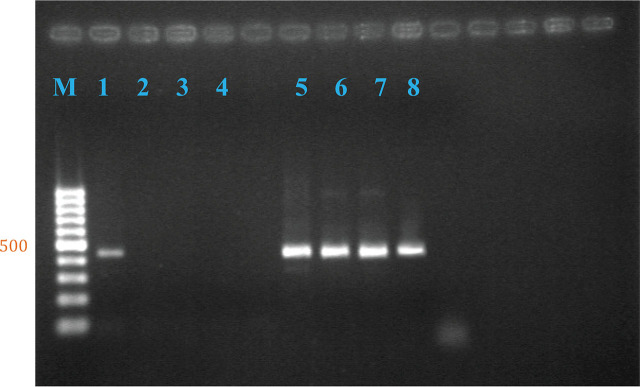
Dengue RNA was detected in the cellular compartment of mobilized bone marrow-derived hematopoietic stem cells by RT-nested PCR targeting the dengue envelope region. The RT-nested PCR products were separated on 2% agarose gels along with the DNA marker indicated on the left side in base pairs (M). Lane 1 = mobilized hematopoietic stem cells, lane 2 = cell-free compartment (supernatant), lane 3 = cell pellet fraction, lane 4 = negative control. Lanes 1–4 are all from blood collected after G-CSF administration; dengue RNA was not detected in any fractions from pre-G-CSF blood (data not shown). Lanes 5–8 = dengue serotypes 1–4, respectively, as positive controls. G-CSF, granulocyte colony-stimulating factor; PCR, polymerase chain reaction; RT, reverse transcription.

For dengue genome detection, PBSC separation was performed on both blood samples by standard gradient isodensity centrifugation. For each sample, cells were obtained both from the interfaces, considered as the mobilized stem cell (MSC) population, and from bottom pellets, considered as mostly erythrocytes, thrombocytes, and nonviable leukocytes. The cell-free supernatant was also collected and analyzed. A total of 6 fractions from the 2 samples were all used for dengue genome detection. RNA was extracted from each fraction, and dengue serotype-specific RT-nested PCR was performed, as described earlier [5]. The first PCR primers were designed to amplify all serotypes of DENV and the second primer pair for specific amplification of each of the 4 serotypes. Genome of dengue serotype 2 was detected in the MSC fraction in both the first round and the nested reaction but was not detected in any of the other 5 fractions. The experiment was carefully repeated 3 more times and was consistently reproducible (**Figure 1**). To avoid contamination, each PCR reaction was performed following the guidelines described earlier [6] (**Table 1**).

**Table 1.** Detection of dengue viral RNA in various fractions of blood obtained before and after G-CSF injection to mobilize hematopoietic stem cells into peripheral blood

	**Post-G-CSF blood fractions**			**Pre-G-CSF blood fractions**		
	**CF**	**MSC**	**CP**	**SR**	**PBMC**	**CP**
Dengue envelope-specific RT-PCR	Neg	Pos	Neg	Neg	Neg	Neg

BMCs, blood mononuclear cells; CF, cell-free fraction; CPs, cell pellets; G-CSF, granulocyte colony-stimulating factor; MSCs, mobilized stem cells; PBMCs, peripheral blood mononuclear cells; RT-PCR, reverse transcription polymerase chain reaction; SR, serum.

The post-G-CSF blood specimen was gradient-centrifuged and separated into three compartments: supernatant cell-free fraction (CF), interface MSCs, and bottom cell pellets (CP). Dengue envelope genome was detected only in the MSC fraction.

The pre-G-CSF blood specimen was gradient-centrifuged and separated into three compartments: supernatant serum (SR), interface peripheral blood mononuclear cells (PBMCs), and bottom cell pellets (CP). Dengue genome was not detected in any fraction.

## Discussion

Certain flaviviruses have been shown to persist in infected hosts. Clinicians are well familiar with hepatitis C virus and its propensity to be associated with chronic hepatitis, cirrhosis, and hepatocellular carcinoma. West Nile virus, a dengue closely related peer flavivirus, has received American and worldwide attention, initially due to its diverse subclinical and clinically variable manifestations and subsequently because of its prolonged urinary excretion for years after acute infection [7]. As the first research group to report the presence of dengue genome in the urine of acutely infected patients [8], we continue seeking further evidence of dengue persistence in human hosts.

Almost all native Thai adults have been infected with dengue virus at least once. This is evidenced by a high seroprevalence (95%) in asymptomatic pregnant women detected by hemagglutination inhibition (HAI) [9]. In addition, more sensitive assays, such as the plaque reduction neutralization test performed in vaccine trials, can detect dengue-specific antibodies in almost all Thai adults. Our present volunteer, who has multiple myeloma in remission, demonstrated detectable dengue immune response by the FRNT, but had clearly negative dengue titers on paired sera by dengue-specific ELISA. This indicates that she had been remotely infected by the virus, likely several years ago, but had not been recently reinfected. Thus, the detected viral genome in PBSCs has likely persisted in her body from the remote past.

A few years ago, our group detected dengue genome in the bone marrow of some patients with hematologic malignancies in remission. The patients did not show serologic evidence of recent dengue infection, which suggests that the virus could have persisted for years after acute infection. In the same way, in the present report, the patient's dengue-specific paired FRNT titers also confirm a remote, and not recent, infection by the virus. We believe that detection of dengue genome in post-G-CSF-stimulated PBSCs of the case is another piece of supportive evidence that dengue does not simply just appear and disappear, at least not in certain infected persons.

The intriguing and worrisome capability of the virus lies in the fact that in the first few days of an acutely febrile patient, it is unpredictable which patient would develop shock syndrome or any other severe manifestations. Moreover, a complete picture of dengue pathogenesis is yet to be elucidated. While almost all severe cases involve those reinfected for the second time with a different serotype, known as secondary infection, over 90% of the secondary cases are not severe. Thus, additional explanation is clearly needed.

Currently, more research is in progress. We hope that other investigators will confirm our finding. Evidence of detecting persistent genome is intriguing as it may provide a new avenue to expand our knowledge of dengue pathogenesis.
